# Core decompression augmented with bone marrow aspirate concentrate for refractory distal sesamoid bone pathology in horses: A retrospective clinical study

**DOI:** 10.1111/vsu.70139

**Published:** 2026-08-01

**Authors:** Grigorios Maleas, Mahmoud Mageed, Kristyna Hargitaiova

**Affiliations:** ^1^ Equuria Orthopedics Emstek Germany; ^2^ Equipixel Veterinary Teleradiology Bakum Germany; ^3^ College of Veterinary Medicine Cornell University Ithaca USA

## Abstract

**Objective:**

To determine short‐ and long‐term outcomes of core decompression combined with autologous bone marrow aspirate concentrate (BMAC) in treating moderate‐to‐severe refractory distal sesamoid bone (DSB) trabecular pathology in horses.

**Study design:**

Retrospective observational study.

**Animals:**

A total of 21 client‐owned Warmblood horses with DSB pathology unresponsive to conservative management.

**Methods:**

Preoperative diagnosis included lameness localization by diagnostic analgesia and magnetic resonance imaging (MRI) confirmation of DSB trabecular pathology. Horses underwent arthroscopic core decompression via the palmar recess of the distal interphalangeal joint followed by intraosseous BMAC injection. Outcomes were assessed clinically and by follow‐up radiography and MRI.

**Results:**

No major intraoperative or postoperative complications occurred in any of the 21 operated horses. Nine horses met inclusion criteria for long‐term follow‐up, which included clinical, radiographic, and MRI assessments at 6 months and up to 18 months postoperatively. At 6 months, six of nine horses (67%) were sound and returned to training. MRI follow‐up demonstrated reduced short tau inversion recovery (STIR) hyperintensity and increased T1‐weighted signal within the DSB trabecular bone, consistent with a reduction in active trabecular bone pathology. Three horses remained lame with unchanged MRI findings. At 18 months, six horses remained sound, and three returned to their previous or intended performance levels. Radiographic remodeling of cyst‐like lesions was evident in three cases.

**Conclusion:**

Core decompression augmented with BMAC was well tolerated with no unexpected complications in this cohort. This approach allowed horses with advanced DSB trabecular pathology refractory to conservative therapy return to function.

**Clinical impact:**

This combined surgical‐orthobiologic approach may offer a viable therapeutic option for horses with advanced refractory navicular bone pathology.

**Abbreviations**
AAEP: American Association of Equine Practitioners
APS: autologous protein solution
BMAC: bone marrow aspirate concentrate
CaD: calcium dobesilate
CD: core decompression
CSL: collateral sesamoid ligament
DDFT: deep digital flexor tendon
DFTS: digital flexor tendon sheath
DIPJ: distal interphalangeal joint
DSB: distal sesamoid bone
FSE: fast spin echo
GRE: gradient recall echo
IOP: intraosseous pressure
IRAP: interleukin‐1 receptor antagonist protein
MRI: magnetic resonance imaging
NB: navicular bursa
NSAID: nonsteroidal anti‐inflammatory drug
OA: osteoarthritis
OCLL: osseous cyst‐like lesion
RLP: regional limb perfusion
SI: signal intensity
STIR: short tau inversion recovery
TA: triamcinolone acetate
WTS: Wehrli traction shoes

## 1 INTRODUCTION

Navicular disease is one of the most common causes of lameness in horses. It affects predominantly the front limbs and numerous anatomical structures can be affected.^1^, ^2^ A recent large‐scale publication has shown lesions in the trabecular bone of the distal sesamoid bone (DSB) as the most prevalent abnormality in horses with foot pain, detected by magnetic resonance imaging (MRI).^3^ The most significant diagnostic factor in DSB pathology seen in MRI is increased signal in short tau inversion recovery (STIR) fat phase, which has been associated with cancellous bone pathology. This results in bone marrow edema and consequently in increased intraosseous pressure (IOP).^4^ Increased IOP has been proposed as a major contributor of lameness in navicular disease characterized by trabecular bone pathology of the DSB.^4^, ^5^ However, navicular disease is a heterogeneous condition with multiple pathogenetic pathways and the relative contribution of IOP to lameness can vary between cases.

Similar osseous pathology and clinical symptoms have been identified in human orthopedic patients.^6^, ^7^ Lesions such as osteonecrosis and bone marrow edema are regarded as a negative prognostic factor and conservative therapy (e.g., bisphosphonates, vasodilators, non‐steroidal anti‐inflammatory drugs, rehabilitation) tends to be unsuccessful in these cases.^6^, ^8^, ^9^ Surgical treatment, termed core decompression (CD), consists of drilling into the necrotic lesion in order to decrease local intraosseous pressure and stimulate neovascularization. The technique alone has been widely used with reasonable success; however, recent literature reports improved surgical outcomes of CD combined with orthobiologics, such as autologous bone marrow derivatives. This combination seems to bring significantly lower pain scores, higher joint functionality and lower numbers of total joint replacements compared to CD alone, particularly in the early stage of bone disease.^6^, ^7^, ^8^, ^9^

Limited literature is available on the use of CD in equine navicular disease. One study utilized a CD approach through the distal interphalangeal joint (DIPJ) in healthy horses and reported short‐term IOP reduction with a level of neovascularization.^5^ No long‐term lameness monitoring was performed in this study; the horses were euthanized at 3 months postoperatively. A more recent study performed osteostixis of the DSB via bursoscopy and combined with postoperative local corticosteroid administration.^10^ This technique seemed to improve lameness, nevertheless new deep digital flexor tendon (DDFT) tears were observed in some of the operated limbs at a 6‐month postoperative follow‐up. Long‐term outcomes of core decompression (CD), alone or combined with orthobiologic therapy, for distal sesamoid bone (DSB) pathology in horses have not been reported. Therefore, the objective of this retrospective clinical study was to describe short‐ and long‐term postoperative clinical outcomes and MRI findings in Warmblood horses with primary trabecular DSB pathology treated with CD augmented by autologous bone marrow aspirate concentrate (BMAC).

## 2 MATERIALS AND METHODS

### 2.1 Case selection

This was a retrospective observational study. Medical records from a single referral orthopedic practice were reviewed to identify horses treated between 2022 and 2025 for lameness localized to the foot. Inclusion criteria were: (1) Non‐racing Warmblood sport horses with uni‐ or bilateral lameness localized to the foot by diagnostic analgesia. (2) MRI demonstrating trabecular bone pathology of the DSB as the primary lesion (Figure 1). (3) Failure of conservative management—defined as persistent lameness despite prolonged treatment (9–32 months), including combination of orthopedic shoeing, rest, medical therapy (e.g., non‐steroidal anti‐inflammatory drugs [NSAIDs], bisphosphonates), and intra‐articular or intrathecal treatments, without return to intended athletic function. (4) Treatment by CD combined with autologous BMAC. (5) Availability of postoperative clinical and MRI follow‐up. Exclusion criteria were: (1) Primary soft tissue pathology of the navicular apparatus (e.g., moderate to severe DDFT lesions). (2) Osseous pathology outside the DSB (e.g., cyst‐like lesions of the distal or middle phalanx). (3) Additional postoperative therapies beyond BMAC and (4) severe hoof malformation. All pre‐ and postoperative MRI examinations were evaluated by board‐certified radiologists (MM and other radiologists) who were blinded to clinical outcomes, including lameness status at follow‐up. Interobserver agreement was not formally assessed. Orthopedic diagnostics and surgical procedures were performed by the same surgeon (GM).

**FIGURE 1:**
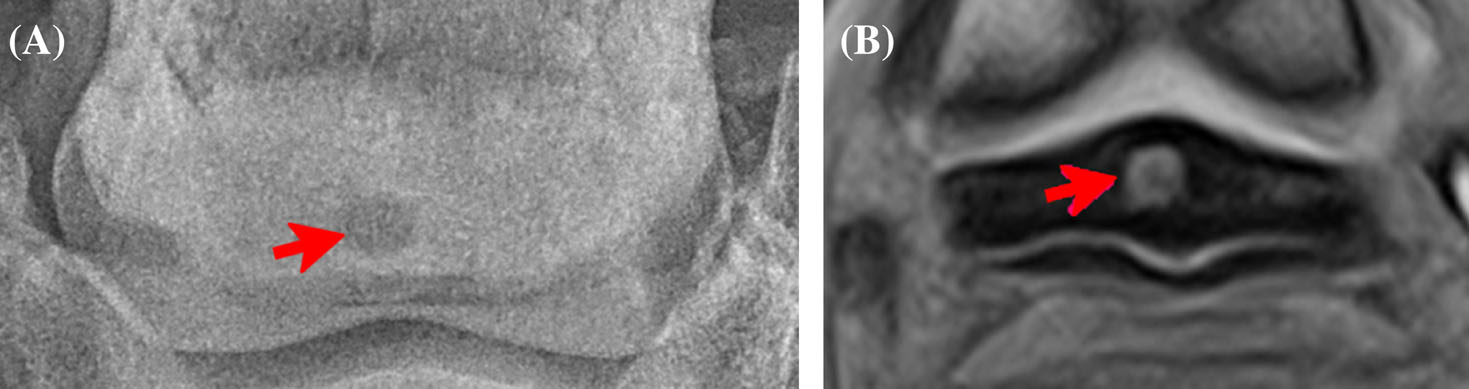
Preoperative imaging of distal sesamoid bone (DSB) pathology. (A) Dorsoproximal 65° palmarodistal oblique radiograph of the navicular bone showing a focal central resorptive lesion in the distal aspect (arrow). (B) Corresponding transverse T1‐weighted magnetic resonance imaging (MRI) showing a central distinct focal heterogenous hyperintense region bordered by diffuse sclerosis, consistent with advanced resorptive trabecular pathology.

### 2.2 Diagnostic approach

A standardized orthopedic examination was performed in all horses. History was recorded, hoof testers used, and the distal limb palpated. Horses were evaluated in motion at walk and trot on a straight line and lunge, on both hard and soft surfaces. Distal and proximal limb flexion tests were performed.

Perineural analgesia – mepivacaine, 1 mL per side (Intra‐Epicaine, Dechra Aulendorf, Germany) was administered using palmar digital nerve blocks, followed—if incomplete—by basisesamoid nerve blocks. After analgesia, standard radiographic projections (lateromedial, dorsoproximal‐palmarodistal oblique, and skyline) were acquired using a portable radiographic unit. Ultrasonography of the distal limb and foot was performed using a 7.5‐MHz microconvex probe via a suprasesamoidean approach.

Horses were subsequently referred for a standing MRI (0.27 T, Hallmarq Veterinary Imaging, Guildford, UK) 2–3 days after diagnostic analgesia. Gradient recall echo (GRE), fast spin echo (FSE), and short tau inversion recovery (STIR) sequences were obtained in sagittal, transverse, and frontal planes. MRI served to confirm DSB trabecular bone pathology and to exclude soft‐tissue‐dominant lesions.

### 2.3 Surgical technique

All procedures were performed under general anesthesia in dorsal recumbency. Bone marrow aspirate (30–60 mL) was harvested from the sternum prior to the procedure and concentrated using a previously described technique.^11^

A standard distal transthecal approach was used to access and evaluate the navicular bursa^12^ and to treat adhesions or fibrillated areas of the DDFT when present. A small abaxial and lateral incision (<10 mm, #15 scalpel) was made into the dorsal aspect of the cul‐de‐sac pouch of the digital flexor tendon sheath (DFTS) to establish the primary viewing portal. The T‐ligament was sharply divided at its closest attachment to the DDFT, and the incision was extended distally to access the NB. To access the DIPJ, the T‐ligament was subsequently divided at its attachment to the palmar surface of the distal P2 and the incision extended proximally toward the coffin joint, as previously described.^13^ Through the lateral viewing portal, a standard 4 mm, 30° arthroscope (Arthrex Inc., Naples, Florida) was introduced and advanced through the DFTS into the palmar recess of the DIPJ, as previously described.^13^

The medial instrument portal was then established under combined arthroscopic guidance. A 21 gauge needle was introduced palmar to the neurovascular bundle with its tip positioned at the proximal surface of the DSB, defining the instrument portal entry point. Under combined arthroscopic and radiographic guidance, the optimal drilling angle and instrument insertion point in the proximodorsal DSB margin were identified using 20 gauge needles (7.5 cm) (Figure 2). A 3.3‐mm guide pin was inserted through a T‐handle, 8 gauge open‐tip decompression needle toward the targeted DSB lesion (Figure 2A,B). The guide pin typically aimed toward the margin of the proximal navicular collateral ligament insertion in the DSB. The pin was further advanced into the lesion and positioning was confirmed using lateromedial and 45°dorsoproximal‐palmarodistal radiographic views.

**FIGURE 2:**
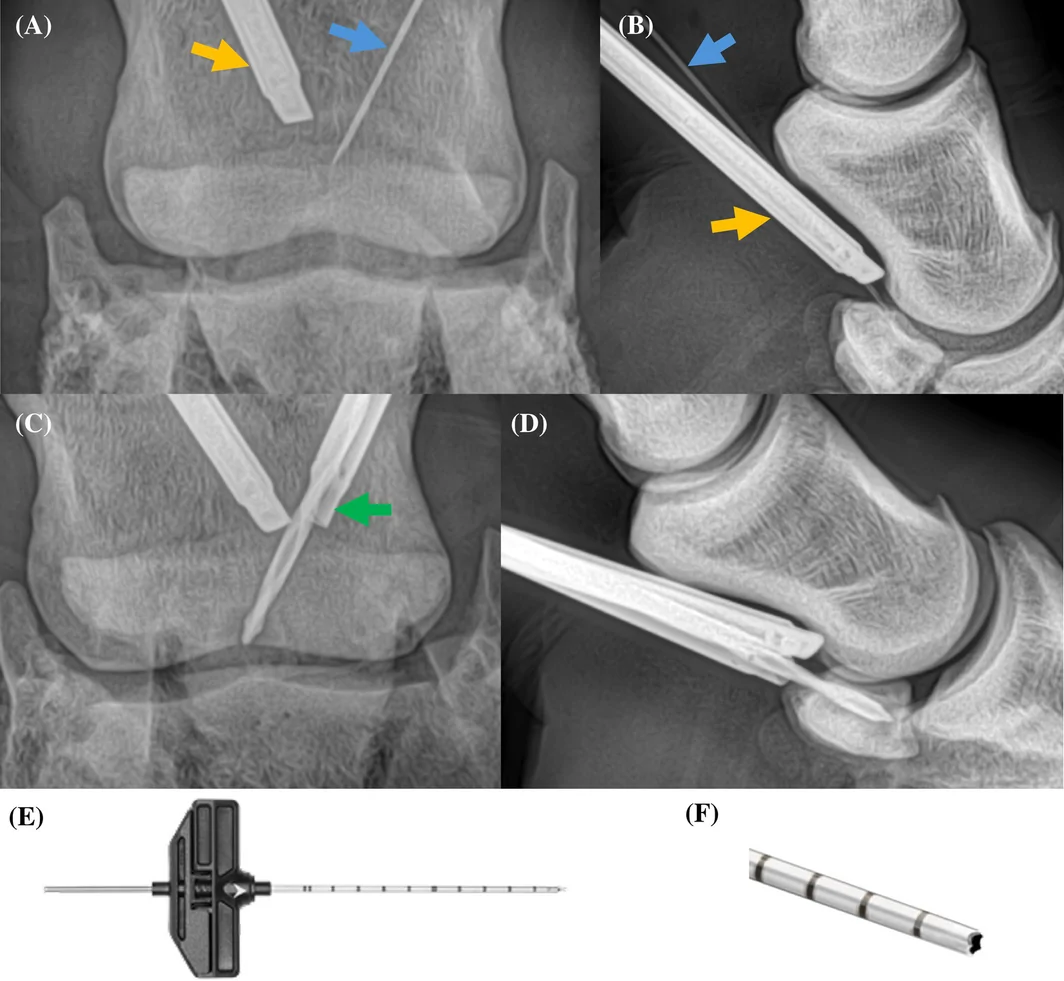
Intraoperative radiographic and arthroscopic guidance during core decompression of the distal sesamoid bone (DSB). (A–B) Intraoperative radiographs (dorsoproximal 45° palmarodistal oblique and lateromedial projections) illustrating optimization of the drill trajectory. A 3.3 mm guiding pin (blue arrow) is inserted through the navicular bursa and directed toward the distal margin of the proximal insertion of the navicular collateral ligament under combined arthroscopic (orange arrow) and radiographic control. (C–D) Intraoperative radiographs (same projections) showing decompression of the resorptive lesion with a drill (green arrow) under arthroscopic and radiographic guidance. (E–F) Arthrex IntraOsseous BioPlasty decompression needle with T‐handle, 13 gauge, 11 cm, open‐tip.

The guide pin was removed and a 3.3 mm, 15 cm bone drill was introduced along the same tract (Figure 2C,D, Figure 3A–C). Targeted lesions included cyst‐like defects, the deepest enlarged synovial invagination or the axial distal third of the DSB in cases of diffuse increased STIR signal. The DIPJ was then thoroughly lavaged, a 13 gauge, 11 cm open‐tip cannulated decompression needle (Arthrex IntraOsseous BioPlasty System, Arthrex Inc., Figure 2E,F) was advanced and retained manually at the most proximal site of the drill channel and the fluid pump was turned off. The decompression needle allowed simultaneous mechanical decompression of the intraosseous lesion and injection of orthobiologic material through the cannulated shaft. Approximately 5–10 mL of BMAC was injected intraosseously using a luer‐lock syringe until overflow was observed at the entry site (usually after ~1–2 mL). The needle was removed, and skin portals were closed in a routine manner. As the drill channel communicates directly with the navicular bursa through the palmar recess of the DIPJ, intraosseous BMAC injection inherently resulted in concurrent bursal exposure; intraosseous and intrabursal administration were therefore not recorded as separate interventions. Remaining BMAC was injected into the dorsal pouch of the DIPJ. In all horses with contralateral DIPJ or navicular bursa pathology, these structures also received BMAC. A sterile distal limb bandage was applied. Recovery was assisted. A radiographic control of the DSB was performed immediately after the recovery phase. No antimicrobials were administered pre‐ or postoperatively.

**FIGURE 3:**
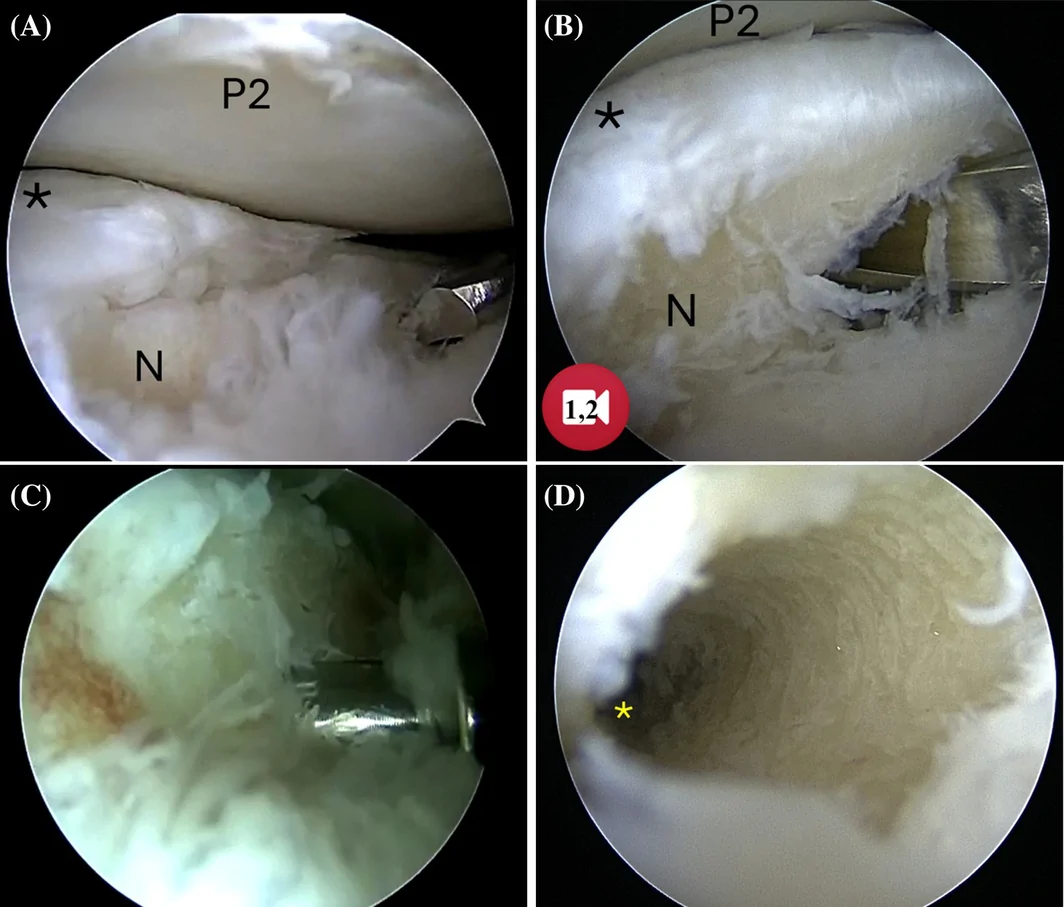
Intraoperative arthroscopic views. (A) Positioning of the drill bit in the distal sesamoid bone (DSB) in palmar recess of the DIPJ. (B, C) Drilling under arthroscopic visualization. (D) Drill channel of the DSB. At the bottom of the channel the cyst lesion can be partially visualized (yellow star). DIPJ, distal interphalangeal joint; N, navicular bone; P2, pastern bone; black star, sagittal ridge of the DSB.

The term “core decompression” is used here in accordance with established orthopedic terminology to describe drilling into affected bone with the aim of reducing IOP and promoting revascularization^6^, ^9^; however, IOP was not directly measured in the present study.

### 2.4 Postoperative management and rehabilitation

Multimodal postoperative analgesia consisted of a cyclooxygenase (COX)‐preferential NSAID meloxicam (Metacam, Boehringer Ingelheim, Ingelheim am Rhein, Germany, 0.6 mg/kg, IV once daily for 2 days) and a non‐NSAID non‐opioid analgesic metamizole (Vetalgin, MSD Animal Health, Boxmeer, Netherlands, 30 mg/kg, IV every 12 h for 3 days) based on their partially distinct mechanisms of action and described clinical use in perioperative pain management in human surgery. Sutures were removed 12 days postoperatively.

In all cases included in this series, individualized shoeing was implemented based on hoof conformation and biomechanical considerations. Owners were advised to maintain the prescribed shoeing protocol on a long‐term basis, as part of lifelong management.

Rehabilitation included 2 weeks of box confinement, 6 weeks hand walking 30 min or 2 × 15 min daily, 6 weeks riding‐walking and 6 weeks gradually increasing interval trotting – 1 min trot, 1 min walk, in total starting on 5 min trot per day on soft surface. Horses gradually resumed full training at approximately 5 months postoperatively, at which time training management was modified in all cases. Adjustments aimed to maintain general fitness while reducing overall workload and discipline‐specific mechanical demands. Specifically, the intensity and frequency of jumping exercises were reduced, and structured recovery days were incorporated, resulting in a training frequency of two to three sessions per week.

### 2.5 Follow‐up examinations and outcome

Follow‐up evaluations were performed at approximately 6 months postoperatively and included clinical examination by the surgeon (GM) and MRI re‐evaluation. Clinical outcomes were corroborated by referring veterinarians and owners. Additional follow‐up at 12–18 months was obtained through in‐person clinical examinations or telephone interviews with owners/riders and referring veterinarians, who were aware of the treatment. Extended follow‐up beyond this point was available for some horses and is reported descriptively. Clinical outcomes were assessed at the level of the horse, while imaging findings were evaluated at the level of the affected limb. Return to previous performance level was defined as return to intended athletic function or training level as reported by owners/riders and referring veterinarians.

### 2.6 Data analysis

This was a descriptive study. Clinical, surgical, and imaging variables were summarized using counts, percentages, means, and SDs where appropriate. Imaging outcomes were qualitatively assessed by comparing pre‐ and postoperative radiographs and MRIs, with emphasis on lesion signal characteristics, synovial effusion, and structural remodeling of the DSB. A Kaplan–Meier curve was generated for descriptive visualization of time‐to‐event data; no inferential statistics were applied due to the small sample size and retrospective design.

## 3 RESULTS

A total of 21 Warmblood horses underwent combined CD with BMAC. Short‐term outcomes were available for the full cohort (*n* = 21). A total of 12 horses were excluded from long‐term analysis due to lack of MRI follow‐up, receipt of additional therapies, or the presence of major pre‐existing soft tissue pathology of the navicular apparatus (e.g., collateral sesamoidean ligament desmopathy), to ensure a more homogeneous cohort for long‐term evaluation. Nine horses met all inclusion criteria and were available for long‐term evaluation (6–18 months postoperatively). These nine horses contributed 11 affected forelimbs (two horses bilaterally, seven unilaterally). Table 1 summarizes signalment and clinical features, and Table 2 outlines diagnostic findings.

**TABLE 1:** Signalment and history of horses.

Horse	Sex	Age (years)	Breed	Athletic use	Owner's complaint/history	Problem duration (months)	Prior treatments	Last preoperative Tx	Interval since last Tx (mo)
1	Gelding	10	Oldenburg	Dressage^LL^	Short stride in trot, heavy in the hand	9	CS & HA, Tildren, NSAIDs	Arthramid, OsPhos	6
2	Gelding	13	Oldenburg	Dressage^LL^	Not willing to work, heavy in the hand, lame	23	CS & HA, NSAIDs, CaD	TA, OsPhos	4
3	Stallion	8	Hanoverian	Jumping^ML^	Landing always left	32	CS & HA, NSAIDs, APS	IRAP, NSAIDs	5
4	Stallion	11	Hanoverian	Jumping^HL^	Lame	18	CS & HA, NSAIDs	TA, NSAIDs	3
5	Gelding	6	Belgian Warmblood	Eventing**	Heavy in the hand left, landing always right, inadequate speed at the end of cross‐country	19	CS & HA, OsPhos	Arthramid, OsPhos	5
6	Gelding	4	Holsteiner	Jumping^ML^	Very lame	12	CS & HA	OsPhos	6
7	Mare	9	Hanoverian	Dressage^LL^	Lame	24	CS & HA, NSAIDs	TA, NSAIDs	4
8	Mare	12	Oldenburg	Dressage^ML^	Lame	11	CS & HA	APS	8
9	Mare	11	Hanoverian	Dressage^HL^	Lame at extended trot	27	CS & HA, NSAIDs, Tildren RLP	Arthramid, OsPhos	4

*Note*: Signalment, athletic use, presenting complaint, lameness duration, prior and most recent treatments, and intervals since last therapy before core decompression + BMAC.

Abbreviations: APS, autologous protein solution; BMAC, bone marrow aspirate concentrate; CaD, calcium dobesilate; CS, corticosteroids; HA, hyaluronic acid; HL, high level; IRAP, interleukin‐1 receptor antagonist protein; LL, lower level; ML, mid‐level; NSAIDs, non‐steroidal anti‐inflammatory drugs; RLP, regional limb perfusion; TA, triamcinolone acetate; Tx, treatment.

**TABLE 2:** Preoperative clinical, radiographic and treatment history.

Horse	Hoof conformation	Shoeing	Gait (AAEP)	Flex test	Rx findings	MRI findings
1	Low heels	Onion shoes	LF: 2/5	++	LF: DSB: lollipop‐shaped synovial invaginations, DIPJ: OA	LF: DSB: moderate altered SI, flexor cortex erosions, NB: effusion, moderate enlarged synovial invagination, DIPJ: mild OA with effusion.
			RF: 2/5	++	RF: DSB: cyst lesion	RF: DSB: OCLL and severe altered SI (STIR FSE), NB: effusion and synovial proliferation, DDFT: mild dorsal fibrillation, DIPJ: moderate synovitis and effusion.
2	Low heels	Full‐roll straight‐bar	LF: 2/5	++	LF: DSB: enlarged synovial invaginations	LF: DSB: moderately altered SI in STIR, enlarged synovial invaginations, DIPJ: moderate effusion DIPJ
			RF: 3/5	++	RF: DSB: CSL entheseopathy, enlarged synovial invaginations	RF: DSB: moderately altered SI in STIR, mild CSL entheseopathy, enlarged synovial invaginations, DIPJ: moderate synovitis and effusion
3	Narrow	Egg bar	3/5	+	DSB: Cyst lesion, DIPJ: mild OA	DSB: diffuse increased signal in STIR, OCLL and flexor cortex erosions in the distal 1/3, NB: synovitis, DIPJ: moderate OA and synovitis
4	Narrow	Full‐rocker	3/5	+++	DSB: enlarged synovial invaginations, DIPJ: OA	DSB: diffuse moderate altered SI in STIR, enlarged synovial invaginations, DIPJ: moderate OA and synovitis
5	Narrow	Wide wedge rolling shoes	2/5	+	DSB: enlarged synovial invaginations, cyst lesion, flexor cortex defect	DSB: OCLL with focal moderate altered SI in STIR, loss of flexor cortex distally, enlarged synovial invaginations, DIPJ: effusion and synovitis
6	Low heels	Open heart bar shoe	3/5	+	DSB: cyst lesion	DSB: severe altered SI, oval well‐defined OCLL
7	Narrow	Egg‐bar with cushion	3/5	+++	DSB: cyst lesion	DSB: focal well‐defined OCLL lesion with hyperintensity in STIR, NB: synovitis, effusion, proliferation, DIPJ: synovitis and effusion, DDFT: mild fibrillations
8	Narrow	WTS shoes	3/5	++	DSB: cyst lesion	DSB: OCLL with severe increased signal in STIR, DIPJ: synovitis, effusion
9	Narrow	Equi‐Librium shoes	2/5	+++	DSB: proximodistal elongation, lollipop‐shaped synovial invaginations	DSB: distal third moderate altered SI in STIR, enlarged synovial invaginations, NB: synovitis, effusion with proliferative tissue, DIPJ: synovitis and effusion, DDFT: mild fibrillation

*Note*: Hoof conformation, shoe type, AAEP lameness grade, distal flexion response, radiographic and MRI findings. Flex test, distal flexion test (+ mild response, ++ moderate, +++ marked).

Abbreviations: CSL, collateral sesamoid ligament; DDFT, deep digital flexor tendon; DIPJ, distal interphalangeal joint; DSB, distal sesamoid bone; FSE, fast spin echo; LF, left fore; MRI, magnetic resonance imaging; NB, navicular bursa; OA, osteoarthritis; OCLL, osseous cyst‐like lesion; RF, right fore; Rx, radiographic evaluation; SI, signal intensity; STIR, short tau inversion recovery; WTS, Wehrli traction shoes; Tx, therapy.

### 3.1 Clinical presentation

Eight horses presented with bilateral forelimb lameness, typically worse on a hard surface and with the affected limb on the inside of the circle; one horse had unilateral forelimb lameness. Lameness duration ranged from 16 to 32 months (mean 19 ± 8 months). Eight affected feet showed a narrow hoof conformation, consistent with chronic preferential heel off‐loading.

### 3.2 Radiographic and MRI findings

Six feet had a unilateral cyst‐like lesion within the DSB. The remaining five feet showed increased STIR signal intensity in the trabecular bone (predominantly distal), with or without enlarged synovial invaginations. Concurrent pathology was common: 8/9 horses had bilateral synovitis or osteoarthritis of the distal interphalangeal joint (DIPJ), and 4/9 had navicular bursitis. Radiographically, seven of 11 feet had a 0° palmar angle (Table 2).

### 3.3 Previous treatments

All horses had failed conservative therapy before referral. Prior treatments included systemic bisphosphonates and calcium dobesilate, intrathecal and/or intra‐articular triamcinolone with hyaluronic acid, 2% polyacrylamide hydrogel, and autologous protein solution (APS) (Table 1).

### 3.4 Surgical observations

Surgeries were carried out without major intraoperative and immediate postoperative complications, and all 21 operated horses were discharged from the practice 12 days postoperatively. The medial palmar digital vein was accidentally injured in one foot without obscuring the arthroscopic vision. Navicular bursa abnormalities included adhesions in six out of 11 feet and irregularities of the flexor cortex in nine of 11 feet. Adhesions were removed using an arthroscopic shaver. Mean duration of surgical procedure per foot was 48 ± 12 min. Recovery was uneventful in all 21 horses. The mean number of radiographs taken per foot intraoperatively was 12 ± 3. Eight horses had concurrent bilateral BMAC treatment of both DIPJs.

### 3.5 Clinical outcomes

Five months postoperatively seven out of nine (78%) horses were sound at trot and gradually returned to normal training (Figure 4A). One horse became lame on the operated limb at the end of 6 months postoperatively. The referring veterinarian diagnosed navicular disease based on a positive PD block response and this horse was retired. At 12–18 months, the referring veterinarians reported all remaining six horses sound (Figure 4B). One horse achieved a higher performance level; two horses returned to the same level and three horses performed at lower level than preoperatively. In total, at 18 months 6/9 (67%) of horses were sound and three of nine horses (33%) achieved same or higher performance level (Figure 4C, Table 3).

**FIGURE 4:**
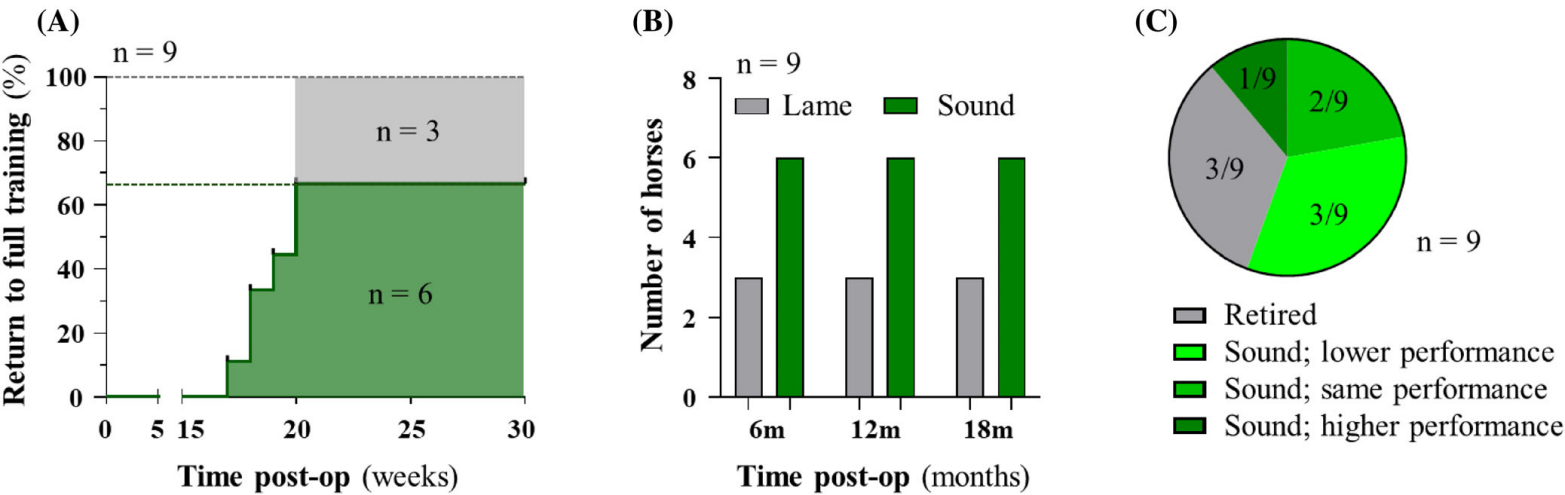
Postoperative performance. (A) A Kaplan–Meier curve is shown for descriptive purposes and illustrates time to return to full training; six horses (67%) achieved training within 18–21 weeks, while three did not resume full work. (B) Proportion of sound (green) and lame (gray) horses at 6, 12, and 18 months postoperatively. (C) Distribution of long‐term performance outcomes: 1/9 horses achieved higher postoperative than preoperative performance, 2/9 horses competed on the same level, 3/9 horses performed on a lower level postoperatively and 3/9 horses were retired.

**TABLE 3:** Clinical and imaging outcomes.

Horse	6 months postoperatively				12 months postoperatively			18 months postoperatively				Interval Sx‐full training (w)
	Gait	Flex	Rx	MRI	Gait	Flex	Rx	Gait	Flex	Rx	Performance	
1	0/5	0	–	LF: signal reduction in STIR	0/5	0	–	0/5	0	↓	Same	18
				RF: DSB: OCLL signal reduction in STIR, NB bursa proliferation without effusion, DIPJ no effusion, DDFT improvement, all other no changes								
2	0/5	0	–	LF: DSB: signal reduction in STIR, DIPJ no effusion, all other no changes	0/5	0	↓	0/5	0	↓	Same	21
				RF: DSB: signal reduction in STIR, DIPJ no effusion, all other no changes								
3	3/5	0	–	DSB: signal reduction in STIR, NB: proliferation, all other no changes	2/5	0	–	2/5	0	↓	x	x
4	0/5	0	–	DSB: signal reduction in STIR, all other no changes	0/5	0	–	0/5	0	–	Lower	20
5	0/5	0	–	DSB: signal reduction in STIR, increased synovial invaginations, DIPJ: reduced effusion, all other no changes	0/5	0	↑	0/5	0	↓	Lower	17
6	2/5	0	–	DSB: moderate signal reduction in STIR	2/5	0	↓	2/5	0	↓	*x*	*x*
7	3/5	0	–	DSB: no change in signal, oval well‐defined OCLL, NB: proliferations, no effusion, DIPJ: no effusion, DDFT improved	2/5	0	Ø	2/5	0	Ø	*x*	*x*
8	0/5	0	–	DSB: signal reduction in STIR, DIPJ normal, all other no changes	0/5	0	↓	0/5	0	↓	Higher	18
9	0/5	0	–	DSB: signal reduction in STIR, NB: proliferation, DIPJ: no effusion, all other no changes	0/5	0	↓	0/5	0	↓	Lower	19

*Note*: Summary of lameness grades, distal limb flexion test response, radiographic (Rx) and MRI changes, and return‐to‐performance outcomes at 6, 12, and 18 months postoperatively. “Interval Sx–full training” indicates the number of weeks between surgery and return to full athletic work.

Abbreviations: –, no radiographic change; ↑, cyst enlarged; ↓, cyst decreased in size; DDFT, deep digital flexor tendon; DIPJ, distal interphalangeal joint; DSB, distal sesamoid bone; Flex, flexion test; Gait, AAEP lameness score; LF/RF, left/right forelimb; MRI, magnetic resonance imaging; NB, navicular bursa; Ø, cyst resolved; OCLL, osseous cyst‐like lesion; Rx, radiographic evaluation; STIR, short tau inversion recovery; Sx, surgery; w, weeks.

### 3.6 Imaging outcomes

At 6 months, there was no radiographic difference in the navicular bone appearance. MRI follow‐up of the sound horses, however, showed a signal reduction in the STIR phase (qualitative decrease in STIR hyperintensity compared to baseline MRI) and signal increase in the T1‐weighted phase of the lesions (Figure 5, Table 3). The remaining three lame horses showed no consistent improvement of the DSB trabecular bone pathology (Figure 6, Table 3). The drill channel was still visible in the navicular bone of all nine horses. All horses showed subjective reduction of synovitis/effusion in DIPJ and navicular bursa at follow‐up MRI. Six out of nine horses were followed further. At 12–18 months, the enlarged invaginations and cyst‐like lesions were still visible on radiographs (Figure 5, Table 3).

**FIGURE 5:**
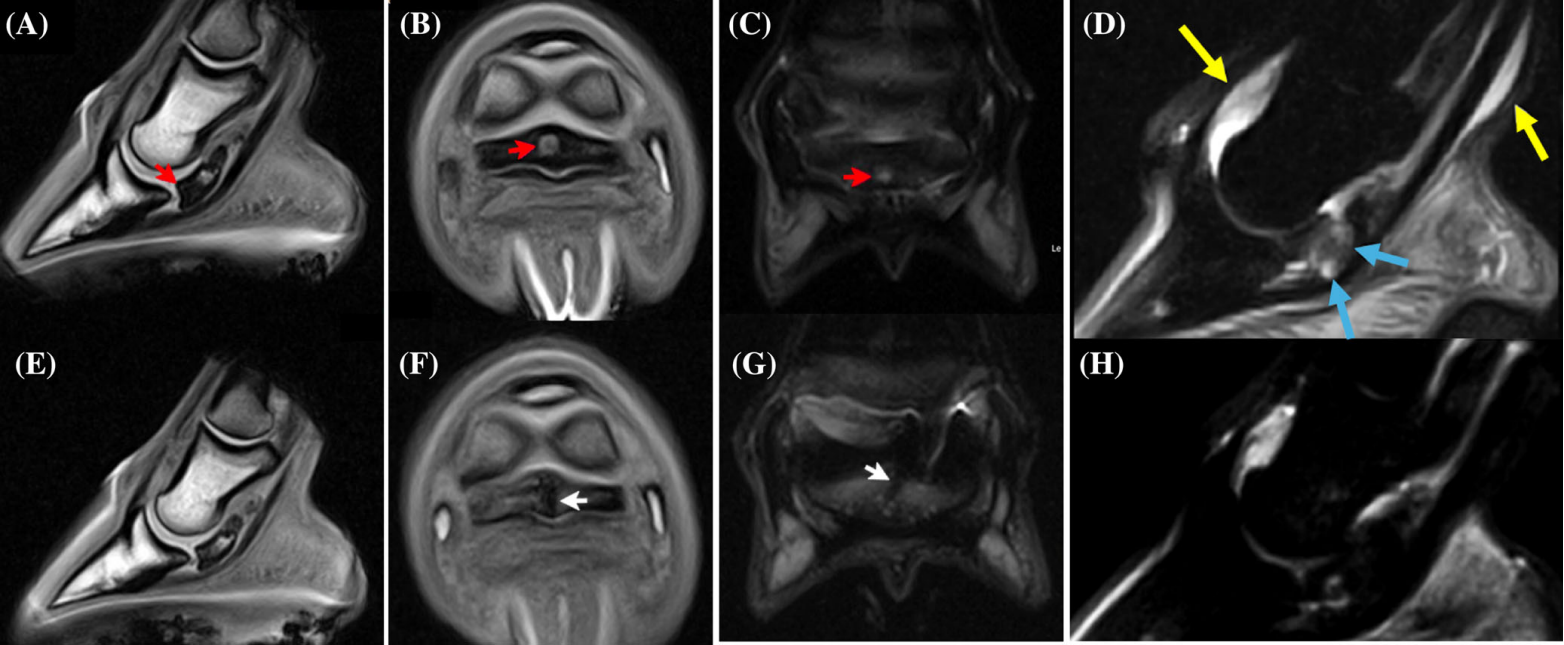
Pre‐ and postoperative magnetic resonance imaging (MRI) changes in a clinically successful case. (A–D) Preoperative standing low‐field MRI: Parasagittal (A) and transverse (B) T1‐weighted spin echo (T1W SE) images show a focal, well‐defined osseous lesion within distal sesamoid bone (DSB) spongiosa (red arrows). (C) Frontal T2‐weighted fast spin echo (T2W FSE) imaging demonstrates marked hyperintensity within the lesion, consistent with resorptive trabecular bone pathology. (D) Sagittal fat suppressed (short tau inversion recovery [STIR]) image shows diffuse moderate heterogeneous hyperintensity accompanied by a focal, spherical lesion in the distal mid‐portion of the DSB (blue arrows). Additional findings include mild effusion of the distal digital flexor tendon sheath (DFTS) and distal interphalangeal joint (DIPJ) (yellow arrows). (E–H) Postoperative MRI at 6‐months follow‐up: Sagittal (E) and transverse (F) T1W FSE sequence shows great resolve of the hyperintensity of the resorptive lesion (white arrow) and improved uniformity of the trabecular architecture. (G) Frontal T2W FSE image demonstrates a substantial reduction in lesion hyperintensity with only mild residual signal. The drill channel is also visible (white arrow). (H) Sagittal STIR image confirms resolution of hyperintense signal in the DSB spongiosa. This horse returned to full athletic function and remained sound at four‐year follow‐up.

**FIGURE 6:**
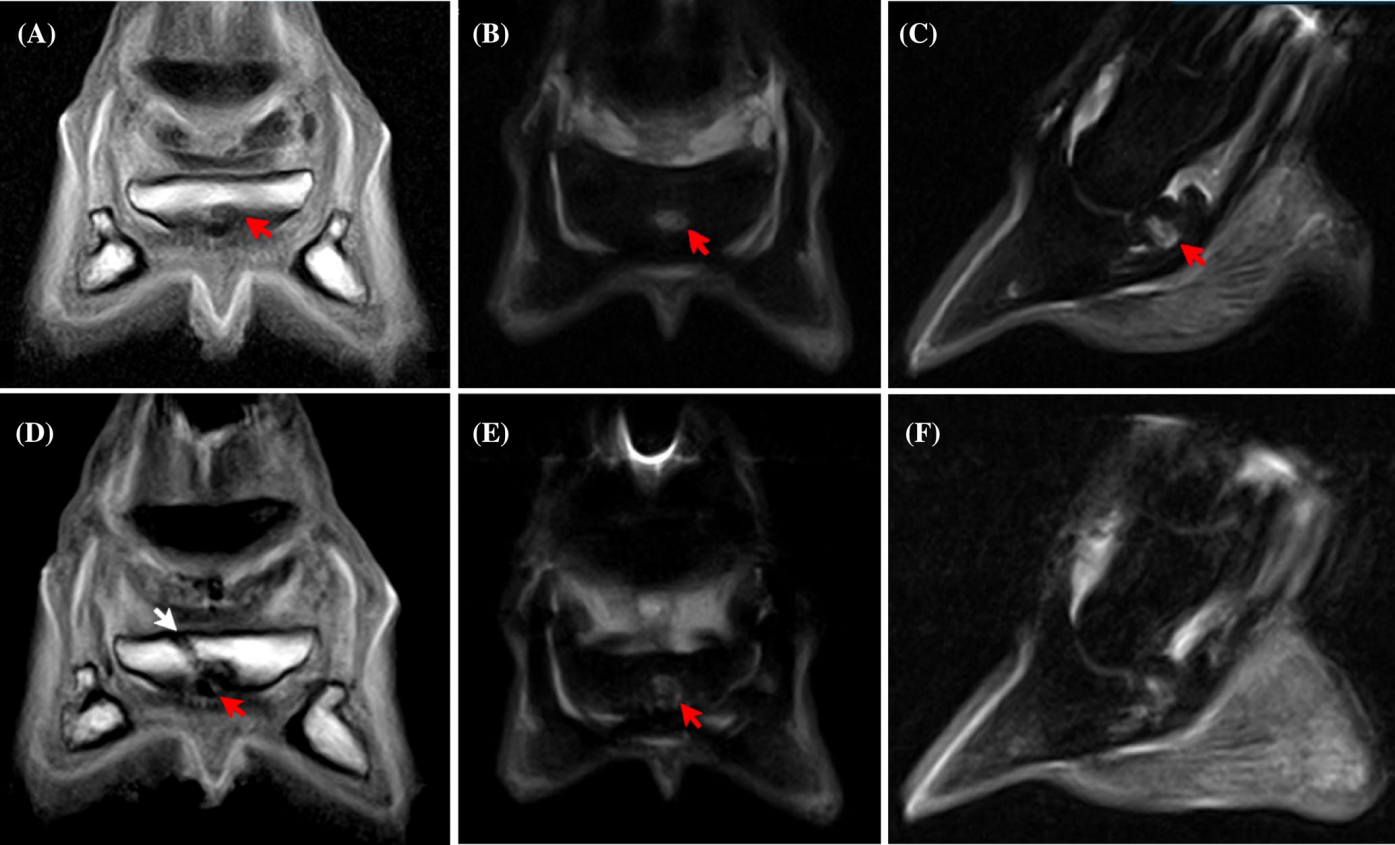
Pre‐ and postoperative magnetic resonance imaging (MRI) changes in a clinically unsuccessful case. (A–C) Preoperative MRI: Frontal T1‐weighted spin echo (T1W SE) (A) frontal (B) and sagittal short tau inversion recovery (STIR) (C) images demonstrate a well‐defined, circular T1 hypointense and STIR hyperintense lesion within the distal sesamoid bone (DSB) spongiosa (red arrows) bordered by a thin sclerotic rim consistent with resorptive trabecular pathology. (D–F) Postoperative MRI (6‐month follow‐up): (D) Frontal T1W SE image shows the osseous lesion still present (red arrow) with the drill channel evident (white arrow). (E–F) Frontal and sagittal STIR sequences show minimal reduction in lesion size or signal intensity (red arrows). This horse remained lame.

## 4 DISCUSSION

This study represents a retrospective evaluation of a clinically indicated surgical procedure performed in a private practice setting. The primary objective was to describe clinical and MRI outcomes in horses with DSB trabecular pathology unresponsive to conservative management. At long‐term follow‐up (12–18 months), six of nine horses (67%) were sound, and MRI re‐evaluation of sound horses demonstrated qualitative reduction in STIR hyperintensity and increased T1‐weighted signal within the DSB trabecular bone; the three horses that remained lame showed no imaging improvement. Although not widely reported, the surgical approach has been previously described in experimental and short‐term clinical settings, supporting its feasibility and safety.^5^, ^10^ No unexpected complications were observed in this cohort.

Consistent with prior reports and based on the authors' experience, conservative management of moderate to advanced DSB pathology generally yields poor long‐term outcomes.^3^ Orthopedic shoeing and rest are most effective in early navicular disease,^14^ while systemic therapies such as bisphosphonates may reduce lameness but do not correct underlying trabecular pathology.^15^ Calcium dobesilate has shown possible benefit,^16^ but interpretation is limited by the absence of controls and the exclusion of horses with concurrent DIPJ or navicular bursa disease, conditions commonly present in clinical cases.^3^, ^17^ Furthermore, intra‐articular and intrathecal therapies have limited efficacy when the primary lesion resides within the DSB spongiosa, likely due to advanced degeneration of cancellous bone.^18^ These observations are consistent with the present cohort, in which all horses had failed prior conservative management before surgical management was selected.

Moderate to advanced navicular disease is histologically characterized by focal or generalized osteonecrosis,^19^ intramedullary hypertension, and venous stasis, all contributing to increased intraosseous pressure (IOP) and pain.^18^, ^20^ Core decompression was developed to reduce IOP and restore perfusion. While early improvements have been reported in human osteonecrosis,^7^, ^9^ long‐term outcomes of CD alone remain inconsistent due to incomplete bone remodeling and insufficient osteogenesis.^9^ Similar limitations have been described in equine studies, where CD alone resulted in only transient reduction in IOP and unsatisfactory clinical results.^5^, ^10^ These findings suggest that long‐term success may depend not only on decompression but also on restoration of normal venous outflow and support of bone regeneration. This pathophysiological framework supports the rationale for the combined approach used in the present study and may explain the clinical improvements observed.

Orthobiologic augmentation has gained interest as an adjunct to CD.^21^ In human orthopedics, BMAC has been used for conditions such as atrophic nonunion and bone marrow edema.^21^, ^22^ BMAC contains concentrated bone marrow mononuclear cells, progenitor cells, cytokines, and growth factors, providing both osteoinductive and osteogenic stimulus. Its paracrine effects and platelet‐mediated angiogenesis may enhance remodeling even in previously avascular tissues.^21^, ^22^ In the present study, BMAC was selected for its osteogenic potential and its modulatory effects on joint inflammation, as well as practicality as an autologous, single‐step therapy suitable for private practice settings. The combined surgical and orthobiologic approach in this study was therefore intended to address both mechanical and biological components of DSB trabecular disease, which may have contributed to the outcomes observed. However, this multimodal approach represents a potential confounding factor and limits the ability to attribute clinical improvement to a single intervention.^23^, ^24^

The combined use of metamizole and meloxicam, although not yet formally evaluated in equine orthopedic surgery, is supported by mechanistic rationale and human clinical data. Meloxicam acts primarily through preferential COX‐2 inhibition, reducing prostaglandin‐mediated peripheral and central sensitization.^25^, ^26^ Metamizole acts as an analgesic drug through a dual mechanism: partial COX inhibition and activation of the endocannabinoid/endovanilloid system, the latter engaging descending pain modulatory pathways not targeted by classical NSAIDs.^27^ This aligns with the principles of multimodal analgesia, which aims to improve pain control through additive or synergistic effects while limiting the dose‐dependent adverse effects of any single drug class.^27^ In human orthopedic surgery, metamizole combined with an NSAID has been shown to reduce postoperative opioid requirements by up to 73% compared to placebo,^28^ and metamizole outperformed paracetamol as an opioid adjunct following total hip arthroplasty.^29^ However, evidence specifically supporting superior analgesia from an NSAID + metamizole combination over monotherapy remains limited, with some studies showing no synergistic effect.^30^ In the present study, the combination was selected based on the authors' clinical experience and the availability of both agents for perioperative IV use in horses. No adverse effects attributable to analgesic administration were observed. Formal pharmacokinetic and pharmacodynamic evaluation of this combination in equine orthopedic patients warrants further investigation.

The study population consisted exclusively of Warmblood sport horses, reflecting the case load at the referral practice. Most horses (8/9) presented with concurrent bilateral DIPJ osteoarthritis and/or navicular bursitis (4/9), a common combination in foot pain.^3^ It is therefore possible that bilateral lameness could have influenced both the clinical presentation and interpretation of lameness, as compensatory gait adaptations can mask or redistribute weight‐bearing asymmetry.^31^ In addition to these clinical considerations, joint effusion, capsular changes, and microcirculatory compromise associated with DIPJ pathology may contribute to secondary remodeling of the DSB and increased IOP.^14^, ^20^ Differentiating pain originating from DIPJ versus DSB lesions remains challenging, and therefore both regions were treated when clinically indicated. Adjunctive use of BMAC for moderate to advanced joint disease is supported by existing equine and human literature and aligns with the authors' clinical experience.^32^

Irrespective of the underlying cause of palmar heel pain, orthopedic shoeing represents a fundamental component of management. All horses in this series were experienced and fully trained; therefore, postoperative training modifications were designed to preserve fitness while reducing mechanical load. Although this study was not designed to isolate the effects of shoeing or training management, these factors may have contributed to the clinical outcomes observed.

Imaging outcomes should be interpreted as complementary to, but not directly equivalent with, clinical improvement. MRI assessment in this study was primarily based on qualitative evaluation of signal changes on STIR sequences. Increased signal intensity on fat‐suppressed images is widely considered to reflect active bone remodeling or edema‐like changes within the DSB and is commonly used in clinical equine MRI to assess disease activity.^4^, ^16^, ^19^ Accordingly, reduction in STIR signal following treatment was interpreted as a clinically meaningful indicator of imaging improvement.^16^, ^18^ While attempts have been made to introduce semi‐quantitative grading systems for DSB pathology, these approaches are not standardized and lack broad consensus or validation for clinical use.^2^, ^4^ Currently, no validated quantitative or semi‐quantitative scoring systems exist for assessment of DSB trabecular pathology using standing low‐field MRI in horses, and qualitative interpretation therefore remains standard in both clinical practice and published literature.^2^, ^3^, ^4^, ^17^, ^18^ Imaging findings therefore represent clinical trends rather than definitive measures of treatment effect.

Further prospective, controlled studies are needed to better define efficacy, prognostic indicators and optimize case selection. In addition, future work should aim to further characterize imaging and biological markers associated with treatment response and to support the development of more standardized outcome measures in clinical practice.

### 4.1 Limitations

This retrospective study and the small number of cases with full follow‐up reflect the constraints of private practice‐based research. As an observational study design, there was no randomization or control group. Clinical follow‐up evaluations were performed by the treating surgeon or house veterinarians who were not blinded. This is a standard clinical practice in equine referral settings where continuity of care is maintained. Outcome measures were based on clinically relevant but partly subjective assessments. These limitations introduce potential sources of bias and preclude definitive causal inference regarding treatment effects.

Quantitative characterization of BMAC (e.g., cell concentration and composition) was not performed, which may introduce variability in the biologic product administered. Horses were not selected based on hoof conformation, despite its known association with navicular disease, and distinguishing primary conformation from secondary changes due to chronic lameness was not possible. Horses with a heel‐offloading gait, which increases load on the navicular apparatus, were also included; trimming and shoeing were managed individually, and no standardized postoperative protocol was applied.

Finally, although MRI was used for follow‐up, restoration of venous perfusion within the DSB could not be confirmed without contrast‐enhanced imaging such as venography or computed tomography (CT).^33^

## 5 CONCLUSION

Core decompression augmented with BMAC may represent a promising treatment option for horses with moderate to severe distal sesamoid bone trabecular pathology unresponsive to conservative management. Clinical improvement observed in this study was accompanied by favorable changes in MRI signal characteristics in most horses. However, given the retrospective design and lack of controls, these findings should be interpreted as descriptive and hypothesis‐generating.

## AUTHOR CONTRIBUTIONS

Maleas G, DVM: Conceptualization (lead), investigation (lead), methodology (lead), project administration (supportive), resources (lead), visualization (equal), writing – original draft preparation (equal), writing – review and editing (equal). Mageed M, Dr. med. vet., Dipl. ECVDI‐Large Animal: Investigation (supportive), visualization (equal), writing – review and editing (supportive). Hargitaiova K, DVM: Data curation (lead), formal analysis (lead), project administration (lead), supervision (lead), visualization (equal), writing – original draft preparation (equal), writing – review and editing (equal).

## CONFLICT OF INTEREST STATEMENT

Mahmoud Mageed: Co‐founder and co‐owner of Equipixel Veterinary Teleradiology. This company had no role in the study design, data analysis, or manuscript preparation. Grigorios Maleas and Kristyna Hargitaiova declare no conflict of interest.

## PRESENTATION OF RESULTS

The results of this study were presented at the American College of Veterinary Surgeons (ACVS) Surgery Summit 2025 in Seattle.

## Supporting information


**Video S1.** Arthroscopic‐guided core decompression of the DSB. Arthroscopic visualization of core decompression of the DSB through the palmar recess of the DIPJ. The drill is advanced into the targeted trabecular lesion while arthroscopy provides direct visualization of instrument placement and protection of the surrounding articular structures.

**Video S2.** Intraosseous BMAC injection following core decompression. Following creation of the decompression channel, a cannulated decompression needle is positioned within the drill tract to deliver autologous BMAC into the trabecular lesion. Overflow at the portal confirms communication with the decompression tract before the remaining BMAC is administered into the DIPJ.

## Data Availability

The data supporting the findings of this study are available from the corresponding author upon reasonable request.
